# Prebiotic inulin ameliorates SARS-CoV-2 infection in hamsters by modulating the gut microbiome

**DOI:** 10.1038/s41538-024-00248-z

**Published:** 2024-03-14

**Authors:** Isaiah Song, Jiayue Yang, Misa Saito, Tenagy Hartanto, Yasunori Nakayama, Takeshi Ichinohe, Shinji Fukuda

**Affiliations:** 1 Institute for Advanced Biosciences, Keio University, Tsuruoka, Yamagata Japan; 2 Metagen, Inc., Tsuruoka, Yamagata Japan; 3 Biolier Business Department, Teijin Limited, Tokyo, Japan; 4 Division of Viral Infection, Department of Infectious Disease Control, International Research Center for Infectious Diseases, Institute of Medical Science, The University of Tokyo, Tokyo, Japan; 5 Gut Environmental Design Group, Kanagawa Institute of Industrial Science and Technology, Kawasaki, Kanagawa Japan; 6 Transborder Medical Research Center, University of Tsukuba, Tsukuba, Ibaraki Japan; 7 Laboratory for Regenerative Microbiology, Juntendo University Graduate School of Medicine, Tokyo, Japan

**Keywords:** Microbiome, Microbiota, Nutrition, Metagenomics

## Abstract

Current treatment options for COVID-19 are limited, with many antivirals and immunomodulators restricted to the most severe cases and preventative care limited to vaccination. As the SARS-CoV-2 virus and its increasing variants threaten to become a permanent fixture of our lives, this new reality necessitates the development of cost-effective and accessible treatment options for COVID-19. Studies have shown that there are correlations between the gut microbiome and severity of COVID-19, especially with regards to production of physiologically beneficial short-chain fatty acids (SCFAs) by gut microbes. In this study, we used a Syrian hamster model to study how dietary consumption of the prebiotic inulin affected morbidity and mortality resulting from SARS-CoV-2 infection. After two weeks of observation, we discovered that inulin supplementation attenuated morbid weight loss and increased survival rate in hamster subjects. An analysis of microbiome community structure showed significant alterations in 15 genera. Notably, there were also small increases in fecal DCA and a significant increase in serum DCA, perhaps highlighting a role for this secondary bile acid in conferring protection against SARS-CoV-2. In light of these results, inulin and other prebiotics are promising targets for future investigation as preventative treatment options for COVID-19.

## Introduction

The spread of coronavirus disease 2019 (COVID-19) has evolved into a global crisis resulting in 768 million cases and almost seven million deaths to date^1^. Vaccine research for SARS-CoV-2, the virus responsible for COVID-19, has long been underway since the advent of the disease. Recent advances in mRNA vaccine technology and an expedited development pipeline led to COVID-19 vaccines being administered as early as December 2020^2^. While this has proven to be effective in reducing rates of infection and mortality^3^, breakthrough cases in vaccinated individuals and instances of severe morbidity necessitate alternative treatment options^3,4^. This is especially critical when considering the emergence of mutant strains exhibiting enhanced immunoevasive capabilities that may reduce the effectiveness of current vaccines^5,6^.

The most severe cases of COVID-19 are characterized by cytokine storm-induced hyperinflammation and are most frequently observed in aging populations^7–9^. Current non-vaccine treatments include antivirals such as Remdesivir that target viral replication machinery^10^ and immunomodulators such as corticosteroids and anti-interleukin-6 antibodies that specifically suppress the exaggerated immune response characteristic of hyperinflammation^11,12^. However, such treatments often carry the risk of severe side effects and are mainly reserved for serious cases or high-risk individuals^13^. These issues necessitate a search for accessible treatments that can ameliorate SARS-CoV-2 infection in a safe, cost-effective manner.

Recovery from COVID-19 is largely dependent on the immune system’s ability to clear the host of the SARS-CoV-2 virus. In principle, supporting the immune system by adopting a healthy lifestyle should also result in improved prognoses, and indeed, certain lifestyle factors such as consumption of a high-fat Western diet, lack of exercise, and sleep deprivation are linked to poor immune function and disease outcomes^14^. Moreover, a study of 592,571 volunteers from the US and UK showed that a high-quality diet consisting of healthy, plant-based foods was associated with both decreased risk and severity of COVID-19^15^, further supporting the notion that a healthy body and immune system are indispensable for positive SARS-CoV-2 infection outcomes.

In addition to maintaining bodily health, it is becoming increasingly known that the trillions of bacteria and other microbes that live within the human gastrointestinal (GI) tract—collectively known as the human gut microbiome—majorly impact host health. The gut microbial community plays important roles in host physiological homeostasis and is strongly implicated in the development and maintenance of a healthy immune system^16^. Probiotics, live cultures of beneficial gut bacteria, and prebiotics, substrates such as dietary fibers and oligosaccharides that are utilized by gut bacteria—recently referred to as microbiota-accessible carbohydrates (MACs)^17^—and are subsequently beneficial to the host, have garnered significant public interest for their purported health benefits. Indeed, probiotics and prebiotics have been reported to be involved in immunomodulation, enhanced nutrient absorption, and strengthening of the gut epithelial barrier^18^. Novel studies have shown that the physiological effects of the gut microbiota are not limited to the GI tract and can be observed in distal organs such as the brain and lungs^19^. Dysbiosis is therefore linked to various diseases ranging from diabetes and non-alcoholic fatty liver disease to atherosclerosis^20^. Furthermore, there is evidence that certain gut microbes and metabolites can potentially attenuate infection by viral pathogens^21^. Studies have shown that disruptions in the gut microbiome and depletion of known immunomodulatory bacteria are associated with severe COVID-19 in hospitalized patients^22,23^. A review of microbiome modulatory studies reported in the literature suggests that prebiotics and other “-biotics” represent a promising avenue through which COVID-19 can be ameliorated^24^.

Inulin is a plant-derived polysaccharide that can be found in many fruits and vegetables but is commonly extracted from chicory root in industrial contexts. It is a soluble fiber prebiotic known to promote growth of probiotic bacteria such as bifidobacteria^25–30^, which have been found to improve prognoses in inflammatory bowel disease, colonic cancers, and *C. difficile* infection^31–33^. In addition, there is evidence that inulin-type fructans promote everyday gut wellness in ways such as increased stool frequency, suppression of pathogenic organism growth, enhanced mineral absorption, and reduced food intake in overweight individuals^34^. These therapeutic effects may largely be driven by production of microbial fermentation end products known as short-chain fatty acids (SCFAs), which are known to confer protective effects on the intestinal environment^31,35,36^ and can modulate the immune system through regulation of T cells and myeloid cells^37–40^. Increased levels of SCFAs as a result of inulin consumption have been well-documented in the literature^30,34,41,42^. Furthermore, inulin-associated SCFAs were shown to boost CD8 + T cell function and protect mice against viral infection^43^, suggesting that inulin may also have the potential to attenuate COVID-19 through SCFAs. From another perspective, inulin supplementation in mice have resulted in increased bile acid circulation and excretion, leading to both negative outcomes such as increased inflammation and liver damage^44,45^ and positive outcomes such as reduced liver fat accumulation in NAFLD^46^. Bile acids are cholesterol-derived molecules produced by the liver that aid in nutrient absorption and are known to play important signaling roles in host physiology. They are able to regulate inflammation via activation of nuclear farnesoid X (FXR) and membrane G protein-coupled bile acid receptor-1 (TGR5)^47,48^ and can be modified by certain intestinal bacteria, facilitating host-microbe interaction^49^. In the literature, certain bile acids were found to reduce cardiac inflammation in mice^50^ and even mitigate the severity of COVID-19 in hamsters^51,52^. Taken together, we hypothesize that inulin may be able to attenuate the symptoms of COVID-19 through mechanisms involving SCFAs and bile acids.

As a widely available prebiotic supplement with documented benefits to the gut microbiome and its host, we therefore decided to explore the potential health benefits of inulin in resisting SARS-CoV-2 infection. In this study, we used a Syrian hamster model to show that dietary inulin supplementation was able to significantly reduce mortality and morbidity caused by SARS-CoV-2 while modulating the gut microbiome composition and metabolite profile. We propose further investigation into inulin and other prebiotics as potential treatment options for suppressing severity of COVID-19.

## Results

### Dietary inulin supplementation improved survival rate and attenuated weight loss in SARS-CoV-2-infected hamsters

SARS-CoV-2 initiates infection of host cells via recognition of angiotensin-converting enzyme 2 (ACE2) receptors expressed on the surfaces of cells in the lungs, vascular endothelium, and intestines^53,54^. However, the receptor-binding domain (RBD) of the SARS-CoV-2 spike protein possesses low binding affinity for mouse and rat ACE2, resulting in poor infectivity^55^. As an alternative animal model for SARS-CoV-2 infection studies, Syrian hamsters are naturally susceptible to the virus and efficiently replicate in the upper and lower respiratory tracts due to their human-like ACE2 structure^56,57^. We therefore utilized this model to study the effects of inulin on COVID-19-induced morbidity and mortality.

**Fig. 1:**
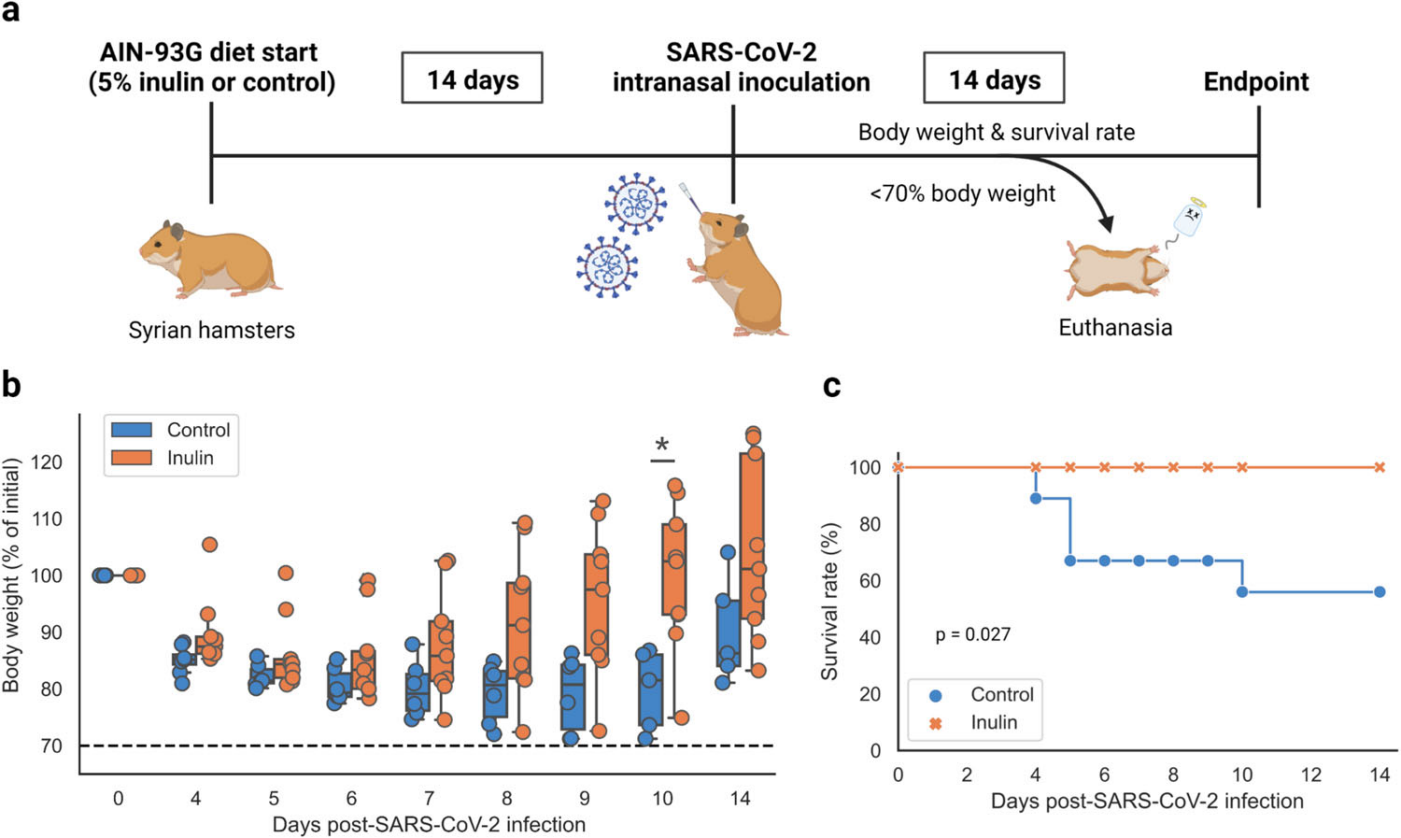
Survival rate and morbid weight loss in SARS-CoV-2-infected hamsters fed a control diet or inulin-supplemented diet. **a** Overview of study parameters. Changes in **b** body weight and **c** survival rate over a two-week period after intranasal infection with SARS-CoV-2 between control (*n* = 9) and inulin-fed (*n* = 9) hamsters. Boxplots denote minimum, maximum, and interquartile range (*p < 0.05). Significance was calculated using the **b** Wilcoxon rank-sum test and **c** log-rank test.

After two weeks of being fed either a control diet or inulin-supplemented diet, hamsters were intranasally infected with SARS-CoV-2 and observed for morbid weight loss and survival rate (Fig. 1a). The survival rate of hamsters infected with SARS-CoV-2 was 100% in the inulin-fed cohort, all of which survived to the end of the two-week post-infection period with no observed weight loss beyond the euthanasia threshold (Fig. 1b, c). Weight loss itself was also attenuated, with five of the nine inulin-fed hamsters retaining >100% of their initial body weight at two weeks and weighing significantly more than control hamsters by Day 10 (Fig. 1b). As a caveat, it is worth noting that the dispersion of body weights within the inulin-fed group was higher than in the control group, appearing to reflect a degree of variability amongst subject responses (Fig. 1b). These trends between the two groups could also be observed to a lesser extent on Days 4 and 9, though just beyond the threshold for significance (*p* = 0.062, 0.054 respectively). In comparison, the mortality rate of the control group was 44%, including one hamster that was euthanized on Day 10 due to excessive weight loss (Fig. 1c). While a pattern of initial weight decline followed by eventual recovery was observable in both cohorts, the inulin-fed hamsters displayed lower weight loss, improved weight recovery, and higher rate of survival.

### Microbiome composition was altered in inulin-fed hamsters

**Fig. 2:**
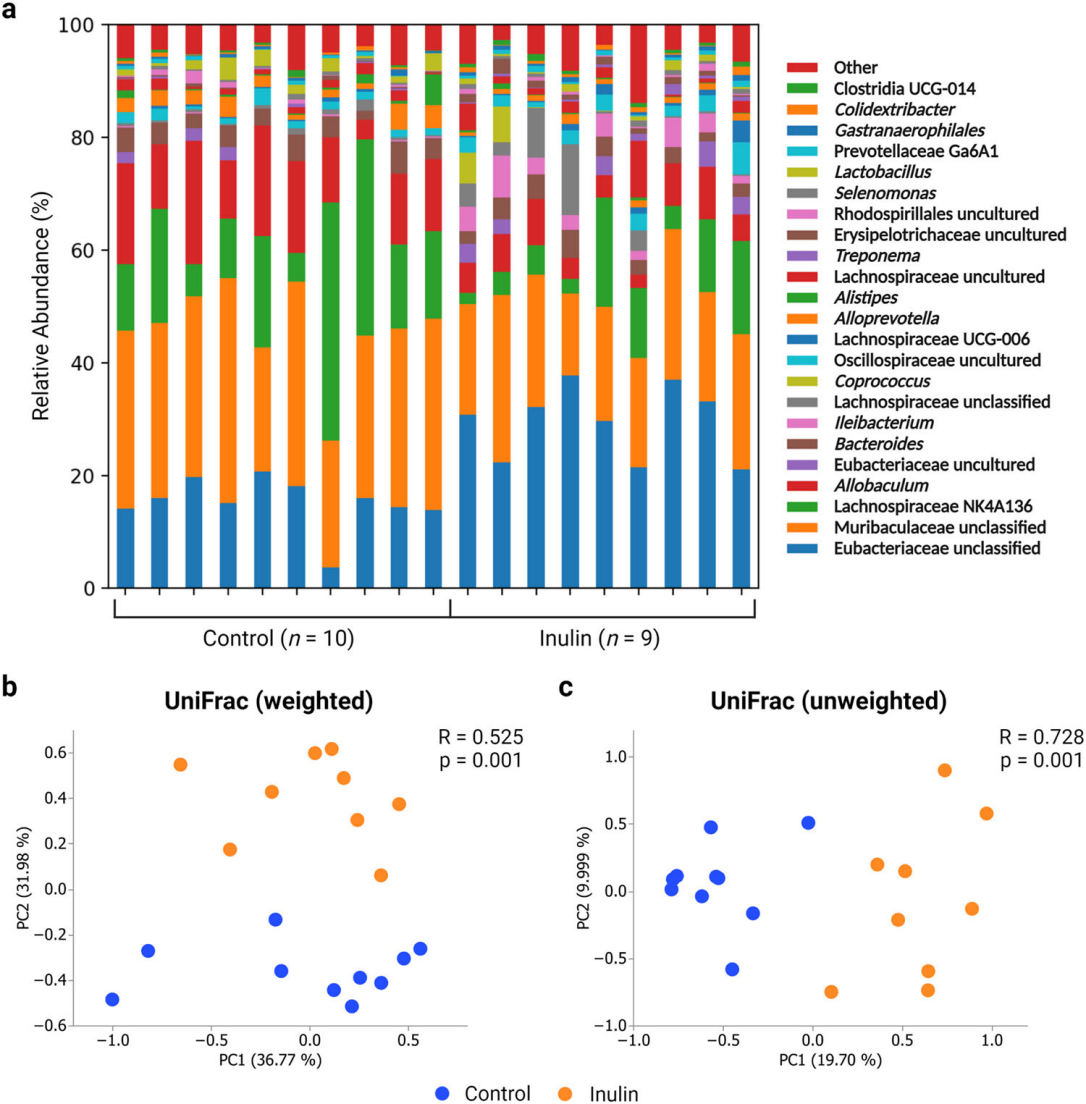
Taxonomy and β-diversity of fecal microbiome communities in Syrian hamsters fed a control diet or inulin-supplemented diet. **a** Relative abundance of genera in fecal samples and **b** weighted and **c** unweighted UniFrac analysis of β-diversity between inulin (*n* = 9) and control (*n* = 10) group microbiome profiles. R and *p* values were calculated using ANOSIM.

**Fig. 3:**
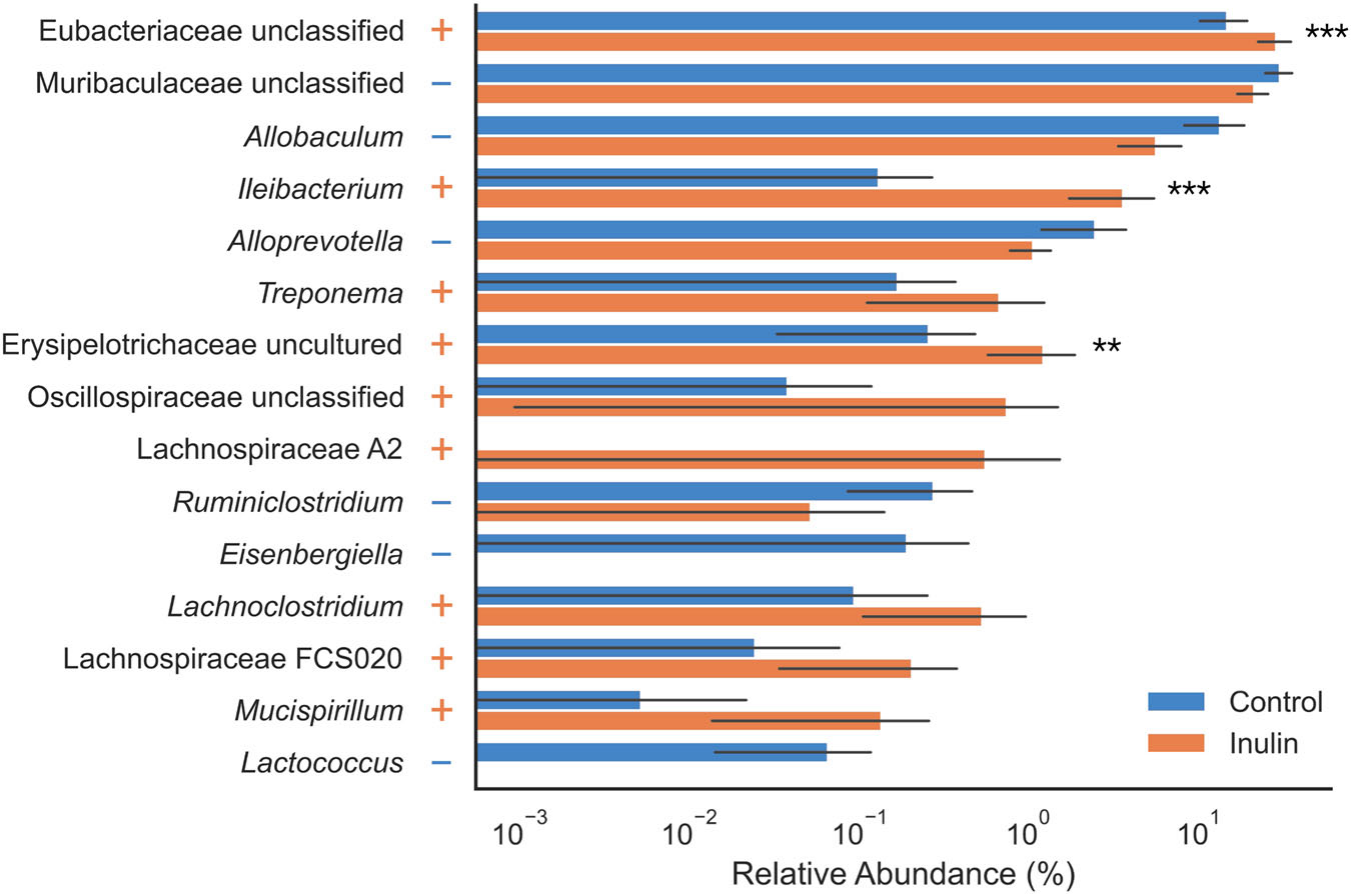
Relative abundances of significantly altered genera in the feces of inulin-fed hamsters. Increased abundance in the inulin group is indicated with a “+”, while decreased abundance is marked with a “-“. Error bars denote standard deviation. Highly significant results as calculated by Wilcoxon rank-sum test are denoted with asterisks (**p < 0.01, ***p < 0.001) and unmarked results reflect *p* < 0.05.

As inulin is best known for its prebiotic effects, we first investigated whether inulin consumption altered microbiome composition as a possible mechanism by which the severity of SARS-CoV-2 infection symptoms was reduced. After two weeks of feeding either a standard control diet or one supplemented with inulin, fecal samples were collected for metagenomic analysis via 16 S rRNA amplicon sequencing. A visual representation of genus-level microbiome composition can be seen in Fig. 2a. The core bacteria comprising the majority of sequence reads across all samples were unclassified members of Eubacteriaceae and Muribaculaceae, as well as Lachnospiraceae NK4A136 and *Allobaculum* (Fig. 2a). However, as can be seen in the UniFrac analysis results, there was significant β-diversity variation between the control and inulin groups regardless of abundance weight (Fig. 2b, c). A total of 15 genera were found to be significantly altered in hamsters fed an inulin-supplemented diet (Fig. 3, Supplementary Table 1). Of these, nine genera increased in abundance while the remaining six decreased. The ASVs classified as *Ileibacterium*, *Mucispirillum*, and an unclassified group of Oscillospiraceae showed the greatest increases with log2 fold changes of 4.79, 4.72, and 4.30, respectively (Supplementary Table 1). The greatest decrease was reflected by *Ruminiclostridium*, with a log2 fold change of -2.41.

### Altered bile acid and SCFA levels in inulin-fed hamsters were correlated with microbial abundance

**Fig. 4:**
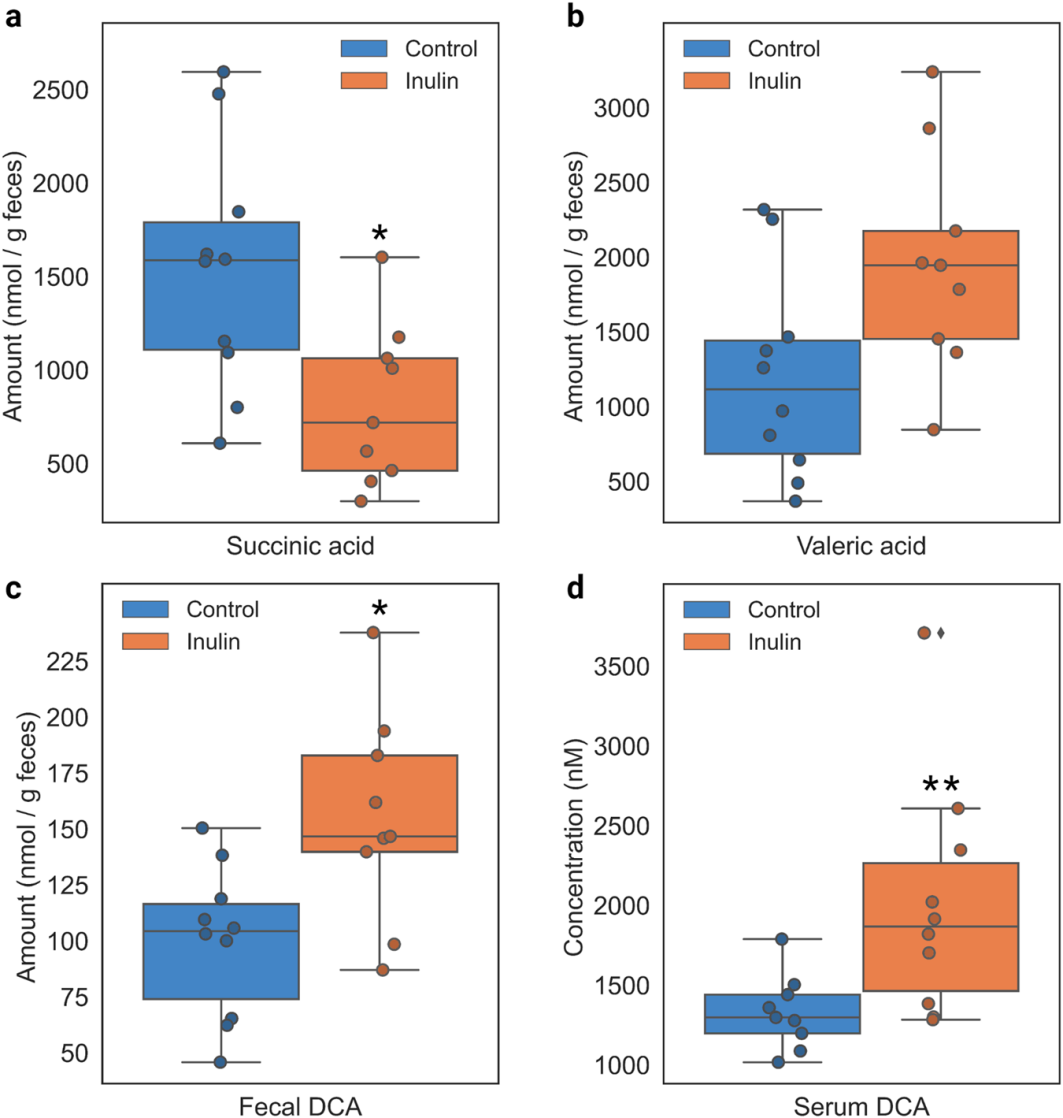
The amounts of SCFAs and bile acids displaying increasing or decreasing trends in inulin-fed hamsters. Boxplots denote minimum, maximum, and interquartile range of **a** fecal succinic acid, **b** fecal valeric acid, **c** fecal DCA, and **d** serum DCA levels (*: pre-FDR-corrected *p* < 0.05, **: pre-FDR-corrected *p* < 0.01). Outliers are marked with a diamond. Significance was calculated using the Wilcoxon rank-sum test.

**Fig. 5:**
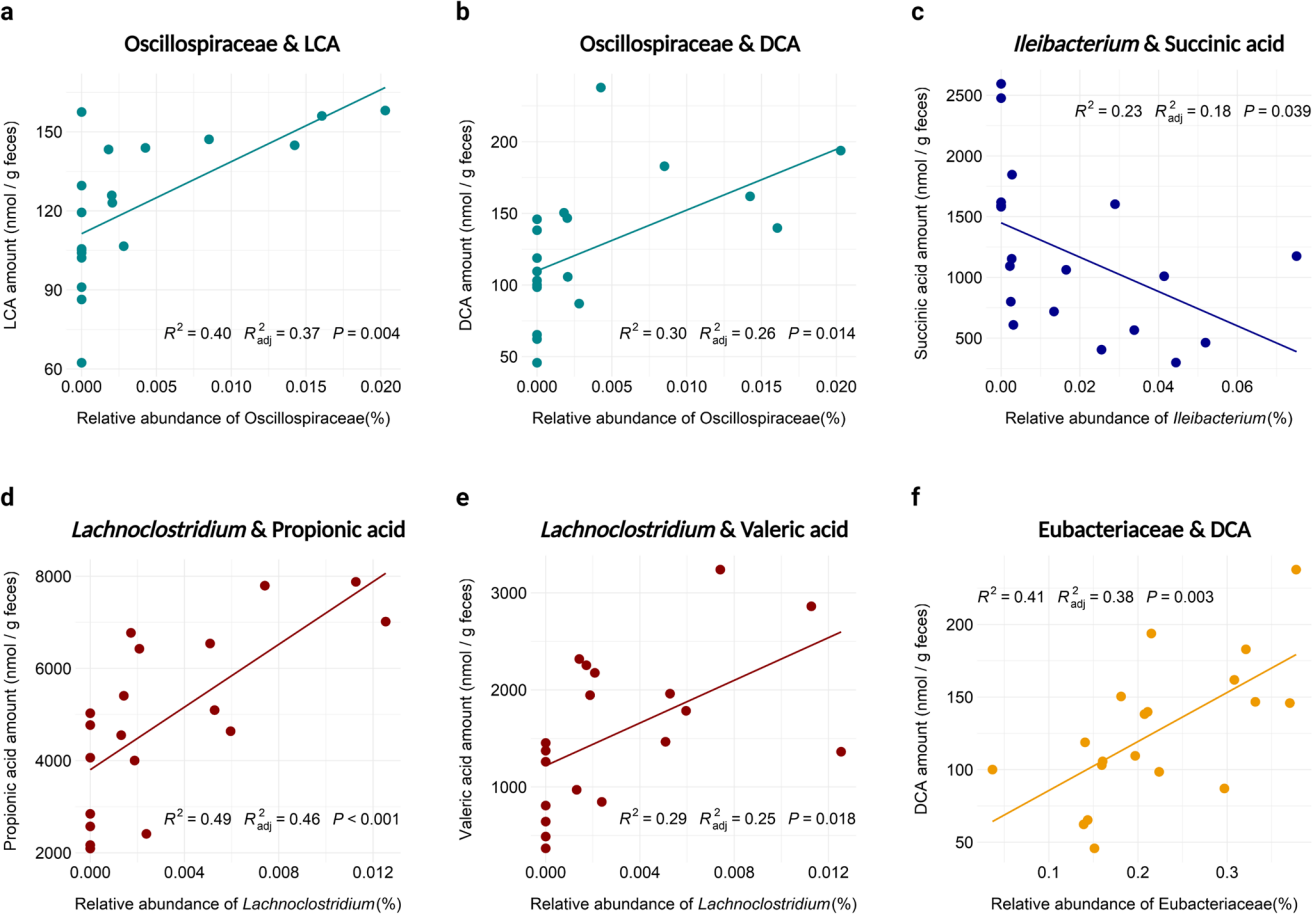
Significant correlations between genera and fecal organic acids in hamsters as a result of dietary inulin supplementation. Correlation plots consist of the genera **a**, **b** Oscillospiraceae unclassified, **c** *Ileibacterium*, **d**, **e** *Lachnoclostridium*, and **f** Eubacteriaceae unclassified. *R*^2^ and *p* values reflect linear regression metrics. Significance was calculated using Spearman correlation.

One of the primary ways that gut microbial communities benefit their hosts is through the production of health-conferring fermentation end-products and other metabolites. Therefore, we analyzed the levels of SCFAs, bile acids, and two other organic acids—lactate and succinate—that are all known to be implicated in host health. Fecal amounts of said metabolites were measured in control and inulin-fed hamster feces after two weeks of feeding (Supplementary Tables 2-3). Additionally, serum samples from a separate cohort of inulin-fed hamsters were analyzed for bile acid content (Supplementary Table 4). However, after FDR adjustment, the only significant change observed in the inulin group was an increase in serum DCA (Fig. 4). Prior to FDR adjustment, we found several other organic acids that were altered either significantly or slightly above the significance threshold of *p* < 0.05: succinate (decreased; *p* = 0.013), valerate (increased; *p* = 0.054), and fecal DCA (increased; *p* = 0.022) (Fig. 4, Supplementary Tables 2-3). However, acknowledging the risk of false positives, we additionally tested for correlative relationships between fecal organic acids and significantly altered members of the microbiota to ascertain whether any of these trends could be supported by correlation data in addition to uncovering any overlooked trends. These analyses did not differentiate between groups and tested purely based on genus and metabolite abundance. Spearman’s rank correlation analysis revealed a total of six significant relationships, including three different genera, three organic acids, and two bile acids (Fig. 5). The results showed that succinate was negatively correlated with relative abundance of *Ileibacterium*, valerate was positively correlated with *Lachnoclostridium*, and DCA was positively correlated with both Oscillospiraceae and Eubacteriaceae (Fig. 4b, c, e, f). Interestingly, these trends were also reflected by the microbiome composition data, in which all four of these genera were more abundant in the inulin group and, following the observed trends, the amounts of their correlated organic acids increased (valerate, DCA) or decreased (succinate) accordingly (Supplementary Tables 1-3). The remaining relationships of Oscillospiraceae and LCA and *Lachnoclostridium* and propionate followed these trends as well (Fig. 5a–d), but changes in LCA and propionate levels were statistically non-significant (Supplementary Tables 2-3). In light of these results, there is evidence that succinate, valerate, and DCA levels were altered in hamsters as a result of dietary inulin supplementation. This is especially conceivable for fecal DCA, as it was already observed that serum DCA levels were significantly increased in inulin-fed hamsters (Fig. 4d, Supplementary Table 4).

## Discussion

The differences in survival rate and weight loss between inulin-fed and control hamsters showed that dietary inulin was able to effectively ameliorate SARS-CoV-2 infection. Furthermore, significant changes in 15 genera as well as increased serum DCA (and less significant changes in succinate, valerate, and fecal DCA) suggest that modulation of the microbiome and its functional characteristics was the driving force behind inulin-induced protection against SARS-CoV-2 infection.

The precise mechanisms by which inulin modulates the gut environment to confer its therapeutic effects may be somewhat ambiguous. There is strong evidence that inulin is digested by members of the gut microbial community, resulting in the enrichment of bacteria that can specifically metabolize these complex carbohydrates, and further cascading into shifts in the microbiome composition based on community dynamics. For instance, butyrate is an important energy source for colonocytes and is implicated in the mitigation of GI inflammation and protection against pathogens^58^. This SCFA has long been recognized for such health benefits and is one of the three most common SCFAs in the human body. Furthermore, other studies have reported that inulin supplementation in rats was associated with a shift in SCFA composition from acetate to propionate and butyrate^59,60^. The butyrogenic effects of inulin appear to be dependent upon cross-feeding interactions between primary inulin degraders and the butyrate-producing beneficiaries that can utilize the resultant secondary metabolites^61^. Going further, such interspecies relationships are certainly dependent on what members are present in the gut microbial community, suggesting that the effects of prebiotics such as inulin—on both the microbiome and the host—may differ based on the individual. Indeed, a study of 174 healthy individuals whose diets were supplemented with one of three different prebiotic fibers (including inulin) showed that changes in SCFA production and gut microbiome composition varied between prebiotics and individuals^61^. It is thus likely that the benefits of prebiotics such as inulin rely on modulation of the gut microbiome by enrichment of host beneficial bacteria, but the mechanistic bases for these benefits and how they would manifest in a given individual are difficult to determine due to the complexity of the gut microbial networks.

Of the 15 significantly altered bacterial genera identified in this study, there was one genus originating from the family Eubacteriaceae and four from Lachnospiraceae (Fig. 3, Supplementary Table 1). These taxa comprise two of the four bacterial families predominantly associated with intestinal butyrate production^59,60^. As such, we expected that an increase in butyrate abundance would be correlated with attenuation of SARS-CoV-2 infection symptoms, especially considering butyrate’s importance in supporting the immune system and intestinal barrier function^58^. However, while we did indeed observe an increase in butyrate, we could not definitively conclude that this change was caused by inulin supplementation due to insufficient statistical power (pre-FDR: *p* = 0.2775) (Supplementary Table 2).

Interestingly, a positive correlation was discovered between *Lachnoclostridium* and propionate (Fig. 5d), both of which were increased in the inulin group (Supplementary Tables 1-2). As propionate is potentially involved in immunoregulatory function^62^, it may have played a protective role against SARS-CoV-2 in this study, though its increase was not significant in inulin-fed hamsters. *Lachnoclostridium* was also positively correlated with the SCFA valerate (Fig. 5e), whose increase in inulin-fed hamsters was just above the threshold for statistical significance (pre-FDR: *p* = 0.0535) (Supplementary Table 2). Unfortunately, the physiological role of microbe-derived valerate is poorly understood, though one interesting study reported that valerate was able to protect GI function and integrity of the intestinal epithelium in irradiated mice^63^, suggesting that it has a protective effect on the GI tract. As for succinate, which was negatively correlated with *Ileibacterium* (Fig. 5c) and was significantly decreased in inulin-fed hamsters (pre-FDR: *p* = 0.0133) (Supplementary Table 2), this organic acid is most often mentioned in reference to diseases such as IBD and obesity, but its overall impact on the human body is relatively unknown^64^. A decrease in succinate may, therefore, be interpreted as an improvement in the general condition of the GI tract in the context of inulin supplementation. *Ileibacterium* is not well-characterized in the literature, but a few studies have shown that species belonging to this genus may be capable of producing SCFAs^65,66^. One possible explanation for its negative correlation with succinate is that it restricts the growth of certain succinate-producing bacteria. Alternatively, it is worth noting that certain gut bacteria can actively convert succinate to propionate^67,68^, which aligns with the trends of decreased succinate and increased propionate observed in inulin-fed hamsters. However, further research is required to conclusively link the changes in microbiome composition to the functional characteristics and resultant fecal metabolite profile that we observed.

And though we can speculate as to why certain microbes and metabolites were altered in response to inulin consumption, it is important to consider the context of the gut microbial ecosystem. In the literature, a study utilizing a “one species out” deletion strategy in a pre-defined, inulin-supplemented bioreactor community of 14 gut microbes reported that when *Lachnoclostridium clostridioforme* was removed from the group, total biomass was reduced in addition to lowering overall inulin consumption^69^. In that same study, *Lachnoclostridium symbiosum* was determined to be the major contributor to butyrate formation, with the abundance of the aforementioned *L. clostridioforme* also determined to be associated with this process by presumably supporting the growth of *L. symbiosum* and other members of the consortium. Such reports demonstrate that even without direct production, certain gut bacteria may indirectly contribute to intestinal metabolite production by influencing other members of the gut microbial community. Our current understanding of gut metabolic networks is insufficient for predicting the cascading effects that will inevitably occur when we attempt to manipulate the gut environment through methods such as prebiotic administration. Additionally, SCFA producers are phylogenetically diverse, making attribution of metabolic function difficult without sufficient evidence such as identification of key metabolic genes or in vitro testing. Taken together, it is clear that the complexity of bacterial metabolic networks, compounded by the polyphyletic nature of SCFA producers and host-microbe metabolic dynamics, necessitates detailed mechanistic studies to elucidate the involvement and role of each SCFA in protection against SARS-CoV-2. Without a more detailed, nuanced understanding of the mechanisms of microbe-microbe and host-microbe interactions, it is difficult to accurately identify and quantify functional characteristics such as SCFA production capacity to such complex communities, necessitating additional mechanistic research on the interactions and functionalities of individual and sub-groups of intestinal microorganisms.

Aside from SCFAs, the bile acid DCA was also shown to be significantly increased in inulin-fed hamster feces (pre-FDR: 0.022) and serum (Fig. 4c, d). DCA is a secondary bile acid that is produced from liver-derived cholic acid (CA) by a small number of gut bacterial species. While 95% of the CA that enters the intestines re-enter hepatic circulation back to the liver, the remaining ~5% are converted into DCA after continuing into the colon and either re-enter hepatic circulation from that point or are excreted^49^. The process of CA-to-DCA conversion is highly efficient, with virtually all of the CA entering the colon being converted into DCA^49^. However, while our study showed a significant increase in serum DCA in inulin-fed hamsters, there were no changes to serum CA levels. A study in the literature showed that inulin supplementation in mice resulted in an overall increase in systemic bile acid levels^44^, which may explain our observations. When considering the bacteria significantly correlated with DCA in our study, only the unclassified Oscillospiraceae and Eubacteriaceae groups were positively correlated with DCA (Fig. 5b, f). However, as there are very few known species capable of converting CA to DCA due to the complex enzymatic requirements of the microbe-mediated Hylemon-Björkhem pathway^70^, it is difficult to determine whether members of these genera are capable of producing DCA. Rather, it is possible that correlations between bile acids and certain species reflect their ability to resist the antimicrobial properties of bile acids, which are particularly potent in secondary bile acids such as DCA^49^. Indeed, bile acid administration has been reported to alter the microbiota composition in rats^71^, suggesting that inulin may indirectly modulate the gut microbiome profile by promoting increased production of antimicrobial bile acids such as DCA.

DCA has primarily been studied in the context of diseases such as colonic and hepatic cancer^72–74^ as a carcinogenic molecule. However, due to its ability to bind the nuclear receptors membrane G protein-coupled bile acid receptor-1 (TGR5) and FXR, which are involved in the regulation of certain inflammatory pathways, it also plays an important role in suppressing inflammation as an agonist for these receptors^75^. In a previous study, DCA was reported to confer resistance to influenza and SARS-CoV-2 infection via immunomodulatory interactions with TGR5 and FXR receptors^52^. Receptor binding by DCA appeared to reduce production of the chemokine CXCL1, preventing chemotaxis and lung infiltration by neutrophils. Neutrophils are a type of granulocyte that eliminates pathogens through the release of cytotoxic antimicrobial agents, which does unfortunately result in “collateral damage” to the surrounding tissues. Consequently, neutrophil accumulation in the lungs and the tissue damage arising from their actions are hallmark symptoms of severe COVID-19^76^. Alternatively, surfactants can act as antiviral compounds through disruption of the viral membrane^77^, and as a digestive surfactant itself, DCA was indeed shown to be able to inactivate the SARS-CoV-2 virus when incubated together at sufficient concentrations^52^. Hence, increased levels of circulating DCA in the body may result in reduced lung inflammation and viral titer due to DCA’s reported ability to regulate the body’s immune response through activation of host cell receptors, and/or inactivating viral particles through direct membrane disruption. However, additional studies are necessary to corroborate these results and clarify the role of DCA and other secondary bile acids in COVID-19 pathogenesis, especially in humans. This is especially true in the context of our study, as it is unclear whether inulin-induced enrichment of DCA was the mechanistic cause of positive disease outcomes.

Another important caveat to consider is that fecal testing may not accurately reflect the fates of intestinal metabolites. To expound, non-insignificant amounts of microbe-derived metabolites are absorbed or degraded by host cells or microbes, thereby affecting their final abundances in excreted feces. This is especially true for SCFAs such as butyrate, which is a common energy source for colonic epithelial cells. The limitation of fecal sampling is the inability to determine microbial production yields from excreted amounts, so the results should be interpreted with these drawbacks in mind. Although there were observable trends in fecal organic acid content that may very well implicate these metabolites in inulin-mediated SARS-CoV-2 resistance, the lack thereof does not necessarily denote irrelevance, and the existence of undetected factors should be considered when interpreting the results of our study.

The dangers of SARS-CoV-2 have diminished since its appearance in 2019, but current trends suggest that this virus will not be eradicated in the near future. It is therefore important for the general public to be aware of ways to mitigate the effects of COVID-19, especially in the case of vulnerable populations. Cheap and widely available prebiotics such as inulin present a promising means for preventing or attenuating SARS-CoV-2 infection while promoting general wellness through the nurturing of a healthy gut microbial community. In our study, we observed that inulin consumption conferred protective effects against SARS-CoV-2 infection in hamsters, demonstrating its therapeutic capabilities. We believe that inulin and other prebiotics represent a promising avenue by which the population can protect itself against COVID-19 and its debilitating effects without the risks of high costs and harsh side effects.

## Methods

### Hamster infection model

All animal experiments using Syrian hamsters were conducted in accordance with the University of Tokyo’s Regulations for Animal Care and Use, approved by the Animal Experiment Committee of the Institute of Medical Science, the University of Tokyo (PA15-92, PA19-87, PA22-33). Four-week-old female Syrian hamsters were purchased from CLEA Japan and divided into two groups with similar average body weights. The control group was fed a standard AIN-93G diet, while the experimental group was fed a 5% (w/w) native chicory inulin (Frutafit® IQ (Sensus B.V., Roosendaal, The Netherlands))-supplemented AIN-93G diet in which the inulin replaced an equivalent amount of the corn starch component by weight. Hamsters were then infected intranasally with 150 µL of a PBS suspension containing SARS-CoV-2 at 10^6^ PFU and were observed for two weeks for the body weight loss and survival rate experiment. In accordance with the proposed animal study plan, subjects that dropped to below 70% of their body weight were humanely euthanized. A total of three different cohorts were utilized for this study, separated into the following three experiments: survival and body weight loss, fecal microbiome and SCFA/bile acid analysis, and serum bile acid analysis. All hamster experiments were conducted in biosafety level three (BLS-3) containment laboratories at the University of Tokyo in accordance with institutional biosafety operating procedures.

### Extraction of metagenomic DNA from fecal samples

Fecal samples were first lyophilized for at least 18 h using a VD-800R lyophilizer (TAITEC CORPORATION, Koshigaya City, Japan). Next, 10 mg of sample were suspended in a mixture of 300 µl of a 10% (w/v) SDS/TE (10 mM Tris-HCl, 1 mM EDTA, and pH 8.0) solution and 300 µl of a phenol/chloroform/isoamyl alcohol (25:24:1) mixture. This suspension was homogenized via agitation with a combination of 3.0 mm and 0.1 mm zirconia beads in a ShakeMaster NEO homogenizer (Biomedical Science, Tokyo, Japan) for 15 min at 1500 × g, and centrifuged at maximum speed for 10 min. A total of 200 µL of the aqueous phase was extracted, from which DNA was then extracted using the GENE PREP STAR PI-480 (Kurabo Industries Ltd., Osaka, Japan) automated extraction system according to manufacturer instructions.

### Metagenomic sequencing and analysis

Microbiome profiling of hamster fecal samples was conducted through amplicon sequencing of the 16 S rRNA gene V1-V2 variable region using the 27 F mod (5'-AGRGTTTGATYMTGGCTCAG-3') and 338 R (5'-TGCTGCCTCCCGTAGGAGT-3') primer set and TKS Gflex DNA polymerase (TaKaRa Bio Inc., Shiga, Japan). After amplification, next-generation sequencing was conducted using Illumina MiSeq (Illumina, Inc., San Diego, CA, USA) and sequence data was analyzed using Qiime2 (version 2021.11). Initial quality filtering and denoising of sequence data was conducted using DADA2 (options: –p-trunc-len-f 280 –p-trunc-len-r 210). Taxonomic assignment was performed using the “feature-classifier classify-sklearn” command with default parameters and Silva SSU Ref Nr 99 (version 138) classifier. Weighted and unweighted UniFrac analyses were conducted using the “diversity core-metrics-phylogenetic” command, followed by analysis of similarities (ANOSIM).

### Quantification of bile acids and SCFAs

Feces or serum were analyzed to identify detectable changes in the composition of bile acids, SCFAs, lactate, and succinate in the case of fecal samples, or bile acids in the case of serum samples. Fecal samples were first lyophilized for at least 18 h using a VD-800R lyophilizer (TAITEC CORPORATION) and were then homogenized by agitating with 3.0 mm zirconia beads for 10 min at 1500 × *g* using a ShakeMaster NEO homogenizer (Biomedical Science). From this point, either 10 mg of the processed fecal sample or 50 µL of serum sample was used for subsequent analyses. Amounts of SCFAs (formate, acetate, propionate, isobutyrate, butyrate, isovalerate, valerate), lactate, and succinate were measured using gas chromatography-mass spectrometry (GC/MS) using previously described methods^78^. Amounts of bile acids were measured using liquid chromatography-mass spectrometry (LC-MS), also using previously described methods^78^.

### Statistical analysis

All statistical analyses were performed in R (version 4.1.0). Significance was calculated using the Wilcoxon rank-sum test and FDR correction in all results unless otherwise noted. Survival rate significance was calculated using the log-rank test in the survival package (version 3.5.5). Correlation was calculated using Spearman’s correlation. Linear regression and significance analysis of correlation data were conducted using the stat_poly_eq function in the ggmisc package (version 0.5.2).

### Reporting summary

Further information on research design is available in the Nature Research Reporting Summary linked to this article.

### Supplementary information

Reporting Summary

Supplementary Tables

## Data Availability

The microbiome data obtained in this study has been deposited in the DDBJ Sequence Read Archive repository under the accession ID: DRA016505.

## References

[1] Geneva: World Health Organization. *WHO COVID-19 Dashboard*. Geneva: World Health Organization. (2023). *WHO COVID-19 Dashboard*. https://Covid19.Who.Int/.

[2] Ball P (2021). The lightning-fast quest for COVID vaccines — and what it means for other diseases. Nature.

[3] Centers for Disease Control and Prevention. Rates of COVID-19 Cases and Deaths by Vaccination Status. *COVID Data Tracker*. https://covid.cdc.gov/covid-data-tracker.

[4] COVID-19 Treatment Guidelines Panel. Coronavirus Disease 2019 (COVID-19) Treatment Guidelines. National Institutes of Health. 34003615

[5] Harvey WT (2021). SARS-CoV-2 variants, spike mutations and immune escape. Nat. Rev. Microbiol..

[6] Gary M (2022). The impact of evolving SARS-CoV-2 mutations and variants on COVID-19 vaccines. mBio.

[7] Tan LY, Komarasamy TV, Rmt Balasubramaniam V (2021). Hyperinflammatory immune response and COVID-19: a double edged sword. Front Immunol..

[8] Brodin P (2021). Immune determinants of COVID-19 disease presentation and severity. Nat. Med..

[9] Merad M, Martin JC (2020). Pathological inflammation in patients with COVID-19: a key role for monocytes and macrophages. Nat. Rev. Immunol..

[10] Gottlieb RL (2021). Early remdesivir to prevent progression to severe Covid-19 in outpatients. N. Engl. J. Med..

[11] van Paassen J (2020). Corticosteroid use in COVID-19 patients: a systematic review and meta-analysis on clinical outcomes. Crit. Care.

[12] Montazersaheb S (2022). COVID-19 infection: an overview on cytokine storm and related interventions. Virol. J..

[13] Sanders JM, Monogue ML, Jodlowski TZ, Cutrell JB (2020). Pharmacologic treatments for coronavirus disease 2019 (COVID-19): a review. JAMA.

[14] Lange KW, Nakamura Y (2020). Lifestyle factors in the prevention of COVID-19. Glob. Health J..

[15] Merino J (2021). Diet quality and risk and severity of COVID-19: a prospective cohort study. Gut.

[16] Belkaid Y, Hand TW (2014). Role of the microbiota in immunity and inflammation. Cell.

[17] Sonnenburg ED (2016). Diet-induced extinctions in the gut microbiota compound over generations. Nature.

[18] Sanders ME, Merenstein DJ, Reid G, Gibson GR, Rastall RA (2019). Probiotics and prebiotics in intestinal health and disease: from biology to the clinic. Nat. Rev. Gastroenterol. Hepatol..

[19] Quinn RA (2020). Global chemical effects of the microbiome include new bile-acid conjugations. Nature.

[20] Fan Y, Pedersen O (2021). Gut microbiota in human metabolic health and disease. Nat. Rev. Microbiol..

[21] Harper A (2021). Viral infections, the microbiome, and probiotics. Front Cell Infect. Microbiol..

[22] Zuo T (2020). Alterations in gut microbiota of patients with COVID-19 during time of hospitalization. Gastroenterology.

[23] Yeoh YK (2021). Gut microbiota composition reflects disease severity and dysfunctional immune responses in patients with COVID-19. Gut.

[24] Xavier-Santos D (2022). Evidences and perspectives of the use of probiotics, prebiotics, synbiotics, and postbiotics as adjuvants for prevention and treatment of COVID-19: a bibliometric analysis and systematic review. Trends Food Sci. Technol..

[25] Hopkins MJ, Macfarlane GT (2003). Nondigestible oligosaccharides enhance bacterial colonization resistance against Clostridium difficile in vitro. Appl. Environ. Microbiol..

[26] Gibson GR, Beatty ER, Wang X, Cummings JH (1995). Selective stimulation of bifidobacteria in the human colon by oligofructose and inulin. Gastroenterology.

[27] Kolida S, Meyer D, Gibson GR (2007). A double-blind placebo-controlled study to establish the bifidogenic dose of inulin in healthy humans. Eur. J. Clin. Nutr..

[28] Ramirez-Farias C (2008). Effect of inulin on the human gut microbiota: stimulation of Bifidobacterium adolescentis and Faecalibacterium prausnitzii. Br. J. Nutr..

[29] Brighenti F, Casiraghi MC, Canzi E, Ferrari A (1999). Effect of consumption of a ready-to-eat breakfast cereal containing inulin on the intestinal milieu and blood lipids in healthy male volunteers. Eur. J. Clin. Nutr..

[30] Birkeland E (2020). Prebiotic effect of inulin-type fructans on faecal microbiota and short-chain fatty acids in type 2 diabetes: a randomised controlled trial. Eur. J. Nutr..

[31] Scharlau D (2009). Mechanisms of primary cancer prevention by butyrate and other products formed during gut flora-mediated fermentation of dietary fibre. Mutat. Res./Rev. Mutat. Res..

[32] Guglielmetti S, Mora D, Gschwender M, Popp K (2011). Randomised clinical trial: Bifidobacterium bifidum MIMBb75 significantly alleviates irritable bowel syndrome and improves quality of life – a double-blind, placebo-controlled study. Aliment Pharm. Ther..

[33] Gueimonde M (2007). Qualitative and quantitative analyses of the bifidobacterial microbiota in the colonic mucosa of patients with colorectal cancer, diverticulitis and inflammatory bowel disease. World J. Gastroenterol..

[34] Schaafsma G, Slavin JL (2015). Significance of inulin fructans in the human diet. Compr. Rev. Food Sci. Food Saf..

[35] McNabney S, Henagan T (2017). Short chain fatty acids in the colon and peripheral tissues: a focus on butyrate, colon cancer, obesity and insulin resistance. Nutrients.

[36] den Besten G (2013). The role of short-chain fatty acids in the interplay between diet, gut microbiota, and host energy metabolism. J. Lipid Res..

[37] Arpaia N (2013). Metabolites produced by commensal bacteria promote peripheral regulatory T-cell generation. Nature.

[38] Maslowski KM (2009). Regulation of inflammatory responses by gut microbiota and chemoattractant receptor GPR43. Nature.

[39] Smith PM (2013). The microbial metabolites, short-chain fatty acids, regulate colonic T cell homeostasis. Science (1979).

[40] Kim CH (2018). Immune regulation by microbiome metabolites. Immunology.

[41] van der Beek CM (2018). The prebiotic inulin improves substrate metabolism and promotes short-chain fatty acid production in overweight to obese men. Metabolism.

[42] Boets E (2015). Quantification of in vivo colonic short chain fatty acid production from inulin. Nutrients.

[43] Trompette A (2018). Dietary fiber confers protection against Flu by shaping Ly6c− patrolling monocyte hematopoiesis and CD8+ T cell metabolism. Immunity.

[44] Arifuzzaman M (2022). Inulin fibre promotes microbiota-derived bile acids and type 2 inflammation. Nature.

[45] Pauly MJ (2020). Inulin supplementation disturbs hepatic cholesterol and bile acid metabolism independent from housing temperature. Nutrients.

[46] Wang R (2022). Inulin activates FXR-FGF15 signaling and further increases bile acids excretion in non-alcoholic fatty liver disease mice. Biochem Biophys. Res Commun..

[47] Gadaleta RM (2011). Farnesoid X receptor activation inhibits inflammation and preserves the intestinal barrier in inflammatory bowel disease. Gut.

[48] Pols TWH, Noriega LG, Nomura M, Auwerx J, Schoonjans K (2011). The bile acid membrane receptor TGR5: a valuable metabolic target. Digestive Dis..

[49] Ridlon JM, Harris SC, Bhowmik S, Kang DJ, Hylemon PB (2016). Consequences of bile salt biotransformations by intestinal bacteria. Gut Microbes.

[50] Wang J (2021). DCA-TGR5 signaling activation alleviates inflammatory response and improves cardiac function in myocardial infarction. J. Mol. Cell Cardiol..

[51] Brevini T (2023). FXR inhibition may protect from SARS-CoV-2 infection by reducing ACE2. Nature.

[52] Nagai M (2023). High body temperature increases gut microbiota-dependent host resistance to influenza A virus and SARS-CoV-2 infection. Nat. Commun..

[53] Hamming I (2004). Tissue distribution of ACE2 protein, the functional receptor for SARS coronavirus. A first step in understanding SARS pathogenesis. J. Pathol..

[54] Jackson CB, Farzan M, Chen B, Choe H (2022). Mechanisms of SARS-CoV-2 entry into cells. Nat. Rev. Mol. Cell Biol..

[55] Wan Y, Shang J, Graham R, Baric RS, Li F (2020). Receptor recognition by the novel coronavirus from Wuhan: ananalysis based on decade-long structural studies of SARS Coronavirus. J. Virol..

[56] Imai M (2020). Syrian hamsters as a small animal model for SARS-CoV-2 infection and countermeasure development. Proc. Natl Acad. Sci. USA.

[57] Chan JF-W (2020). Simulation of the clinical and pathological manifestations of coronavirus disease 2019 (COVID-19) in a golden Syrian hamster model: implications for disease pathogenesis and transmissibility. Clin. Infect. Dis..

[58] Guilloteau P (2010). From the gut to the peripheral tissues: the multiple effects of butyrate. Nutr. Res Rev..

[59] Fu X, Liu Z, Zhu C, Mou H, Kong Q (2019). Nondigestible carbohydrates, butyrate, and butyrate-producing bacteria. Crit. Rev. Food Sci. Nutr..

[60] Vital M, Howe AC, Tiedje JM (2014). Revealing the bacterial butyrate synthesis pathways by analyzing (Meta)genomic data. mBio.

[61] Baxter NT (2019). Dynamics of human gut microbiota and short-chain fatty acids in response to dietary interventions with three fermentable fibers. mBio.

[62] Parada Venegas D (2019). Short chain fatty acids (SCFAs)-mediated gut epithelial and immune regulation and its relevance for inflammatory bowel diseases. Front Immunol..

[63] Li Y (2020). Gut commensal derived-valeric acid protects against radiation injuries. Gut Microbes.

[64] Fernández-Veledo S, Vendrell J (2019). Gut microbiota-derived succinate: friend or foe in human metabolic diseases?. Rev. Endocr. Metab. Disord..

[65] Cabral L (2022). Gut microbiome of the largest living rodent harbors unprecedented enzymatic systems to degrade plant polysaccharides. Nat. Commun..

[66] den Hartigh LJ (2018). Obese mice losing weight due to trans-10,cis-12 conjugated linoleic acid supplementation or food restriction harbor distinct gut microbiota. J. Nutr..

[67] Reichardt N (2014). Phylogenetic distribution of three pathways for propionate production within the human gut microbiota. ISME J..

[68] Flint HJ, Duncan SH, Scott KP, Louis P (2015). Links between diet, gut microbiota composition and gut metabolism. Proc. Nutr. Soc..

[69] Gutiérrez N, Garrido D (2019). Species deletions from microbiome consortia reveal key metabolic interactions between gut microbes. mSystems.

[70] Ridlon JM, Daniel SL, Gaskins HR (2023). The Hylemon-Björkhem pathway of bile acid 7-dehydroxylation: history, biochemistry, and microbiology. J. Lipid Res.

[71] Islam KBMS (2011). Bile acid is a host factor that regulates the composition of the cecal microbiota in rats. Gastroenterology.

[72] Cao H (2017). Secondary bile acid-induced dysbiosis promotes intestinal carcinogenesis. Int J. Cancer.

[73] Yoshimoto S (2013). Obesity-induced gut microbial metabolite promotes liver cancer through senescence secretome. Nature.

[74] McGarr SE, Ridlon JM, Hylemon PB (2005). Diet, anaerobic bacterial metabolism, and colon cancer: a review of the literature. J. Clin. Gastroenterol..

[75] Shulpekova Y (2022). The role of bile acids in the human body and in the development of diseases. Molecules.

[76] Reusch N (2021). Neutrophils in COVID-19. Front Immunol..

[77] Simon M, Veit M, Osterrieder K, Gradzielski M (2021). Surfactants – Compounds for inactivation of SARS-CoV-2 and other enveloped viruses. Curr. Opin. Colloid Interface Sci..

[78] Hashimoto S (2023). Changes in intestinal bacteria and imbalances of metabolites induced in the intestines of pancreatic ductal adenocarcinoma patients in a Japanese population: a preliminary result. Scand. J. Gastroenterol..

