# Supplementary material for: Prebiotic inulin ameliorates SARS-CoV-2 infection in hamsters by modulating the gut microbiome
Source: NPJ Sci Food. 2024 Mar 14;8:18. doi: 10.1038/s41538-024-00248-z (PMC10940623; doi:10.1038/s41538-024-00248-z)
Supplement: Supplementary file 2 — Supplementary Tables [file 41538_2024_248_MOESM2_ESM.pdf]

**Supplementary Table 1. Relative abundances of genera significantly altered in inulin-fed hamsters.**

| Taxon | Control | Inulin | Log2 FC | q-value |
|---|---|---|---|---|
| Eubacteriaceae unclassified | 0.1516 ± 0.0444 | 0.2949 ± 0.0607 | 0.96 | 0.0009*** |
| *Ileibacterium* | 0.0013 ± 0.0013 | 0.0367 ± 0.0180 | 4.79 | 0.0009*** |
| Erysipelotrichaceae uncultured | 0.0026 ± 0.0022 | 0.0125 ± 0.0062 | 2.25 | 0.0025** |
| *Allobaculum* | 0.1377 ± 0.0503 | 0.0577 ± 0.0220 | -1.25 | 0.0224* |
| *Ruminiclostridium* | 0.0028 ± 0.0018 | 0.0005 ± 0.0009 | -2.41 | 0.0224* |
| Lachnospiraceae FCS020 | 0.0002 ± 0.0005 | 0.0021 ± 0.0016 | 3.07 | 0.0224* |
| Muribaculaceae | 0.3098 ± 0.0526 | 0.2190 ± 0.0426 | -0.50 | 0.0239* |
| Lachnospiraceae A2 | ND | 0.0057 ± 0.0093 | N/A | 0.0239* |
| *Eisenbergiella* | 0.0019 ± 0.0024 | ND | N/A | 0.0239* |
| *Lachnoclostridium* | 0.0010 ± 0.0015 | 0.0054 ± 0.0041 | 2.51 | 0.0239* |
| *Lactococcus* | 0.0007 ± 0.0005 | ND | N/A | 0.0239* |
| *Treponema* | 0.0017 ± 0.0019 | 0.0069 ± 0.0054 | 1.99 | 0.0268* |
| Oscillospiraceae | 0.0004 ± 0.0008 | 0.0076 ± 0.0071 | 4.30 | 0.0268* |
| *Mucispirillum* | 0.0001 ± 0.0002 | 0.0014 ± 0.0012 | 4.72 | 0.0333* |
| *Alloprevotella* | 0.0252 ± 0.0124 | 0.0108 ± 0.0028 | -1.22 | 0.0432* |

Significance was calculated using the Wilcoxon rank-sum test. FDR-corrected p values are listed as q values (*: $q < 0.05$, **: $q < 0.01$, ***: $q < 0.001$). Control: $n = 10$ and inulin: $n = 9$.

**Supplementary Table 2. Amounts of fecal SCFAs in control and inulin-fed hamsters.**

| Organic acid | Control (nmol/g) | Inulin (nmol/g) | Log2 FC | p-value | q-value |
|---|---|---|---|---|---|
| Formic acid | 1691.42 ± 731.50 | 2070.42 ± 2106.61 | 0.29 | 0.7197 | 1.0000 |
| Acetic acid | 33672.49 ± 7856.15 | 36023.55 ± 6996.87 | 0.10 | 0.9682 | 1.0000 |
| Propionic acid | 4397.73 ± 1597.17 | 5345.75 ± 1931.84 | 0.28 | 0.3154 | 0.7908 |
| Isobutyric acid | 136.14 ± 32.61 | 155.77 ± 45.55 | 0.19 | 0.4470 | 0.7908 |
| Butyric acid | 5415.49 ± 3633.34 | 6991.90 ± 2985.34 | 0.37 | 0.2775 | 0.7908 |
| Isovaleric acid | 89.88 ± 28.85 | 107.48 ± 37.96 | 0.26 | 0.4470 | 0.7908 |
| Valeric acid | 1195.08 ± 646.54 | 1959.02 ± 697.69 | 0.71 | 0.0535 | 0.40998 |
| Lactic acid | 98.21 ± 44.57 | 129.16 ± 72.02 | 0.40 | 0.4470 | 0.7908 |
| Succinic acid | 1536.60 ± 621.06 | 811.20 ± 405.60 | -0.92 | 0.0133* | 0.2532 |

Wilcoxon rank-sum significance values are listed pre- and post-FDR correction (*: p or q < 0.05). Control: $n$ = 10 and inulin: $n$ = 9.

**Supplementary Table 3. Amounts of fecal bile acids in control and inulin-fed hamsters.**

| Bile acid | Control (nmol/g) | Inulin (nmol/g) | Log2 FC | p-value | q-value |
|---|---|---|---|---|---|
| LCA | 116.215 ± 21.584 | 127.783 ± 29.615 | 0.14 | 0.1823 | 0.6917 |
| UDCA | 0.035 ± 0.106 | 0.032 ± 0.0915 | -0.13 | 1.0000 | 1.0000 |
| CDCA | ND | ND | N/A | 1.0000 | 1.0000 |
| DCA | 99.928 ± 31.711 | 154.923 ± 43.953 | 0.63 | 0.0220* | 0.2532 |
| CA | 5.704 ± 2.329 | 9.871 ± 9.244 | 0.79 | 0.3562 | 0.7908 |
| GUDCA | ND | ND | N/A | 1.0000 | 1.0000 |
| GCDCA | ND | ND | N/A | 1.0000 | 1.0000 |
| GDCA | 0.004 ± 0.007 | ND | N/A | 0.2105 | 0.6917 |
| GCA | ND | ND | N/A | 1.0000 | 1.0000 |
| TLCA | 0.058 ± 0.060 | 0.026 ± 0.050 | -1.17 | 0.1609 | 0.6917 |
| TUDCA | ND | ND | N/A | 1.0000 | 1.0000 |
| TCDCA | ND | ND | N/A | 1.0000 | 1.0000 |
| TDCA | 0.116 ± 0.099 | 0.061 ± 0.118 | -0.93 | 0.1299 | 0.6917 |
| TCA | ND | ND | N/A | 1.0000 | 1.0000 |

Wilcoxon rank-sum significance values are listed pre- and post-FDR correction (*: p or q < 0.05). Control: $n$ = 10 and inulin: $n$ = 9.

**Supplementary Table 4. Concentrations of serum bile acids in control and inulin-fed hamsters.**

| Bile acid | Control (nM) | Inulin (nM) | Log2 FC | p-value | q-value |
|---|---|---|---|---|---|
| CDCA | ND | 1138.07 ± 1761.21 | N/A | 0.2105 | 0.5263 |
| DCA | 1329.94 ± 218.98 | 2009.08 ± 701.32 | 0.60 | 0.0076** | 0.0381* |
| CA | 15220.34 ± 4298.07 | 15878.01 ± 6942.01 | 0.06 | 0.9682 | 1.0000 |
| GCDCA | 39.22 ± 110.94 | 76.44 ± 153.38 | 0.96 | 0.5975 | 0.9959 |
| GDCA | ND | 34.62 ± 103.85 | N/A | 1.0000 | 1.0000 |

Wilcoxon rank-sum significance values are listed pre- and post-FDR correction (*: p or q < 0.05, **: p or q < 0.01). Control: $n$ = 9 and inulin: $n$ = 10.
